# Epidemiology of Pertussis Among Young Pakistani Infants: A Community-Based Prospective Surveillance Study

**DOI:** 10.1093/cid/ciw561

**Published:** 2016-11-02

**Authors:** Saad B. Omer, A. Momin Kazi, Robert A. Bednarczyk, Kristen E. Allen, Conrad P. Quinn, Fatima Aziz, Khurram Sial, Varun K. Phadke, Maria L. Tondella, Margaret M. Williams, Walter A. Orenstein, S. Asad Ali

**Affiliations:** 1 Department of Global Health; 2 Department of Epidemiology; 3 Department of Pediatrics; 4 Department of Emory Vaccine Center, Emory University, Atlanta, Georgia; 5 Department of Paediatrics and Child Health, Aga Khan University, Karachi, Pakistan; 6 Meningitis and Vaccine Preventable Diseases Branch, Division of Bacterial Diseases, National Center for Immunization and Respiratory Diseases, Centers for Disease Control and Prevention; 7 Division of Infectious Diseases, Emory University, Atlanta, Georgia

**Keywords:** pertussis, maternal vaccine, Tdap, Pakistan, surveillance

## Abstract

***Background.*** Pertussis remains a cause of morbidity and mortality among young infants. There are limited data on the pertussis disease burden in this age group from low- and lower-middle-income countries, including in South Asia.

***Methods.*** We conducted an active community-based surveillance study from February 2015 to April 2016 among 2 cohorts of young infants in 4 low-income settlements in Karachi, Pakistan. Infants were enrolled either at birth (closed cohort) or at ages up to 10 weeks (open cohort) and followed until 18 weeks of age. Nasopharyngeal swab specimens were obtained from infants who met a standardized syndromic case definition and tested for *Bordetella pertussis* using real-time polymerase chain reaction. We determined the incidence of pertussis using a protocol-defined case definition, as well as the US Centers for Disease Control and Prevention (CDC) definitions for confirmed and probable pertussis.

***Results.*** Of 2021 infants enrolled into the study, 8 infants met the protocol-defined pertussis case definition, for an incidence of 3.96 (95% confidence interval [CI], 1.84–7.50) cases per 1000 infants. Seven of the pertussis cases met the CDC pertussis case definition (5 confirmed, 2 probable), for incidences of CDC-defined confirmed pertussis of 2.47 (95% CI, .90–5.48) cases per 1000 infants, and probable pertussis of 0.99 (95% CI, .17–3.27) cases per 1000 infants. Three of the pertussis cases were severe according to the Modified Preziosi Scale score.

***Conclusions.*** In one of the first prospective surveillance studies of infant pertussis in a developing country, we identified a moderate burden of pertussis disease in early infancy in Pakistan.

Despite the availability of pertussis vaccines for routine immunization for >6 decades, pertussis remains endemic worldwide. The World Health Organization (WHO) estimates that every year there are approximately 16 million cases of pertussis worldwide, with 95% occurring in developing countries [1]. Infants too young to be completely vaccinated are a high-risk group for acquiring pertussis [2]. However, pertussis burden estimates are based on sparse data from developing countries.

Maternal immunization with tetanus, diphtheria, and acellular pertussis vaccine (Tdap) during pregnancy is a potential strategy to protect young infants against pertussis in 2 ways: First, passively transferred antipertussis antibodies can directly protect the infant and second, prevention of pertussis in the mother can indirectly protect the child through decreasing exposure. Although Tdap is already recommended for pregnant women in the United Kingdom, the United States, Australia, New Zealand, Belgium, Spain, and some Latin American countries, it is not part of routine antenatal care in most developing countries where the burden of pertussis is ostensibly much higher.

We aimed to address a critical knowledge gap in the estimate of incidence and associated morbidity due to pertussis in a developing country setting.

## METHODS

The Prevention of Pertussis in Young Infants in Pakistan (PrePY) Baseline Surveillance study was conducted in 4 low-income settlements of Karachi (Rehri Goth, Ibrahim Hyderi, Bhains Colony, and Ali Akbar Shah) where the Department of Paediatrics and Child Health of The Aga Khan University (AKU) has been running primary healthcare centers (staffed with physicians, lady health visitors, and community health workers) for several years. AKU has an active population based Demographic Surveillance System (DSS) in the study areas with a total surveillance catchment population of approximately 220 000.

Enrollment for this surveillance study in the 4 study sites in Karachi began on 21 February 2015 and the last follow-up occurred on 12 April 2016. Surveillance was conducted among both an open cohort, with infants enrolled at ages up to 10 weeks and followed through 18 weeks of age, and a smaller closed cohort, with pregnant women enrolled on or after 27 weeks’ gestation or mothers enrolled who gave birth within the prior 72 hours; infants born to these women were followed through 18 weeks of age. For both cohorts, infants were routinely evaluated for symptoms associated with a syndromic screening definition (described later), and for infants who met the syndromic screening definition, nasopharyngeal swabs and blood samples were collected for laboratory testing.

**Figure 1.**
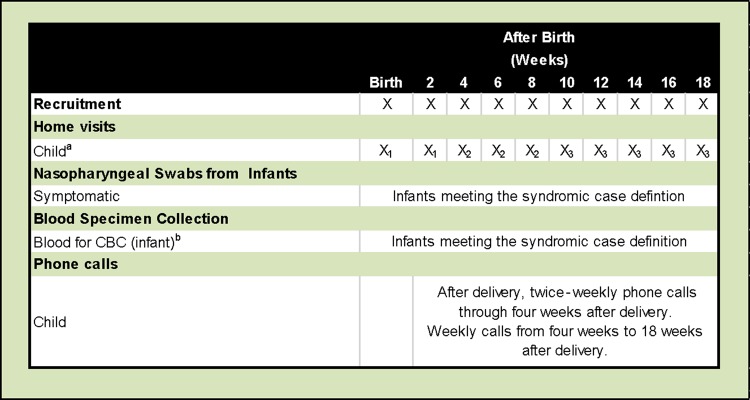
Open cohort study schedule. ^a^Schedule for surveillance visits will be based on the infant's age at enrollment, not time since enrollment; ^b^Three milliliters of blood to be collected for infant specimens. Home visit key: X_1_ = twice weekly; X_2_ = weekly; X_3_ = fortnightly. Abbreviation: CBC, complete blood count.

Infant surveillance occurred through routine scheduled in-person visits, telephone follow-up, and additional unscheduled visits and calls, according to the following schedule (Figure 1): Infant follow-up home visits were made twice a week from birth through 2 weeks of age. From 3 to 7 weeks of age, follow-up home visits occurred once a week; from 8 through 18 weeks of age, follow-up home visits were conducted every 2 weeks. In addition to home visit follow-ups, phone calls were made twice weekly from delivery through 4 weeks of age. Weekly phone calls were made from 4 to 18 weeks of age.

Basic demographic data were obtained from mothers and infants at the time of enrollment, including age at enrollment, anthropometric measurements (infant length, weight, and head circumference), and maternal history of tetanus toxoid (TT) receipt. At all surveillance visits, infants were assessed against the standardized syndromic criteria, which is defined as an infant presenting with any of the following symptoms: cough (lasting at least 1 day), coryza, whoop, apnea, posttussive emesis, cyanosis, seizure, tachypnea (>50 breaths/minute for infants >2 months or >60 breaths/minute for infants <2 months), severe chest indrawing, movement only when stimulated (or an alternative definition of lethargy), poor feeding (confirmed by poor suck), close exposure to any family member with a prolonged afebrile cough illness, or axillary temperature ≥38°C.

Our analysis was based on 2 outcome definitions: (1) The infant met the syndromic definition and had a positive polymerase chain reaction (PCR) test for *Bordetella pertussis*; and (2) the infant met the US Centers for Disease Control and Prevention (CDC) case definition of probable or confirmed pertussis (see Supplementary Data).

To ensure the most accurate clinical description of each case, we identified syndromic symptoms within the time frame from cough onset through diagnosis and any continued symptoms without a break in symptom presentation that would be considered part of that illness episode. To identify new illness episodes as discrete, we required 7 days without symptoms. Because the CDC clinical case definition requires symptom assessment over time, based on a cough with a duration of ≥2 weeks, this approach is in line with the CDC clinical criteria. This provided us the ability to conduct a longitudinal assessment that captured all symptoms within the clinical episode, rather than being limited to a snapshot of symptoms at only 1 visit.

Infants meeting the syndromic screening definition had nasopharyngeal swabs obtained by trained physicians using sterile, individually wrapped Copan FLOQ Minitip Nylon Flocked Dry Swabs. These swabs have comparable performance to rayon swabs [3, 4], and their use is recommended by CDC for optimal specimen collection for PCR testing for *B. pertussis*. To minimize exposure, the physician obtaining the swab wore a surgical mask and clean gloves. Swabs were inserted nasally and advanced along the floor of the nose, until they reached the nasopharynx. Once at the nasopharynx, the swabs were held against the posterior nasopharynx for a few seconds. Swabs were collected and stored in labeled universal transport medium cryovials and transported to the AKU Infectious Disease Research Laboratory at 4°C. Samples were stored at −70°C until total nucleic acid extraction for PCR.

The PCR procedures were in line with the CDC protocols for *B. pertussis* PCR and were adopted in consultation with the CDC [5, 6]. Total nucleic acid was extracted from the frozen aliquots using MagNA Pure Compact Nucleic Acid Isolation Kit I (Roche Life Science, Indianapolis, Indiana). Leftover DNA samples following initial testing were archived at AKU's Infectious Disease Research Laboratory, with storage of at least 2 years at −80°C. DNA extracts were tested by PCR for evidence of *B. pertussis* infection, using a real-time PCR detection system (Applied Biosystems 7500, Thermo Fisher Scientific, Waltham, Massachusetts).

All assays were run with positive and negative controls using standardized preparations of *B. pertussis* DNA, as well as PCR for the RNAse-P enzyme, which is used as a quality control measure to confirm that nasopharyngeal swabs successfully contacted human mucosa during the sampling. In line with the CDC analysis criteria for monoplex real-time PCR, cycle threshold (Ct) values <35 for IS*481* were considered positive for *B. pertussis*, with IS*481* Ct values ≥35 and <40 requiring further confirmation with *ptxS1* testing. We tested all samples positive for IS*481* using the *ptxS1* assay, and *ptxS1* Ct values <40 were considered a positive reaction.

For all infants, accumulated person-time, measured in person-months, was computed, and based on month of enrollment, month-specific person-time accumulated was computed. Overall confirmed pertussis cases were identified by month of occurrence, and calendar month-specific and overall incidence rates with 95% confidence intervals (CIs) were calculated.

We computed the (modified) Preziosi score for pertussis severity based on the presence or absence of specific symptoms, as well as a modified version of the Preziosi scoring system [7] which includes additional symptoms (Supplementary Figure 1). Infants were categorized as having severe pertussis if their Modified Preziosi Scale score was ≥7, and categorized as having moderate pertussis if their score ranged from 1 to 6.

## RESULTS

### Study Population and Descriptive Statistics

Of the 2021 infants enrolled into the surveillance study, 1800 (89.1%) were enrolled into the open cohort, and 221 (10.9%) enrolled into the closed cohort. The total surveillance cohort contained slightly more male than female infants (52.3% vs 47.7%, respectively). Detailed demographics (infant anthropometric measurements and receipt of birth vaccines) are shown in Table 1. Age at enrollment and anthropometric measures were similar among infants who had a positive PCR test for *B. pertussis* compared to those who did not have a positive PCR test for *B. pertussis* (Table 1).

**Table 1.** Baseline Characteristics of Infants

Infant Characteristics and No. of Infants Assessed for Overall Comparison	Overall	Infants Without Positive PCR for *B. pertussis* (n = 2013)	Infants With Positive PCR for *B. pertussis* (n = 8)
Age at enrollment, d, median (IQR) (n = 2017)	20 (9–41)	20 (9–41)	18 (14–26.5)
Weight at enrollment, g, median (IQR) (n = 1900)	3320 (2820–3970)	3320 (2820–3970)	3001 (2600–3350)
Length at enrollment, cm, median (IQR) (n = 1900)	51.5 (49.0–53.9)	51.5 (49.0–54.0)	51.3 (46.3–51.5)
Head circumference at enrollment, cm, median (IQR)	35.3 (33.8–36.5)	35.3 (33.8–36.5)	35.0 (33.0–35.7)
Male sex	52.3%	62.5%	52.2%
Birth weight, g, median (IQR)^a^	2800 (2500–3000)	2800 (2500–3000)	2600 (2600–2600)
Birth immunizations received, No. (%)			
BCG	1706 (84.4)	1701 (84.5)	5 (62.5)
OPV	1705 (84.4)	1700 (84.5)	5 (62.5)

Abbreviations: *B. pertussis*, *Bordetella pertussis*; IQR, interquartile range; OPV, oral polio vaccine; PCR, polymerase chain reaction.

^a^ Specific to infants enrolled in the closed cohort (n = 221) only. Note that only 1 infant in the closed cohort had a positive PCR test for *B. pertussis*.

Of the 8 infants with positive pertussis tests, all met our protocol-defined initial pertussis case definition, namely, they met the syndromic screening definition and had a positive PCR test for pertussis. Of these 8 infants, 7 met the CDC pertussis case definition (5 met the criteria for CDC confirmed pertussis cases, 2 met the criteria for CDC probable pertussis cases); only 1 of these 8 did not meet the CDC pertussis case criteria, as this infant did not have cough, with syndromic screening identifying only coryza and chest indrawing. A total of 1311 infants met the syndromic screening definition, of whom 1303 (99.4%) did not have PCR-positive tests.

The incidence of pertussis per 1000 infants according to our pertussis case definition was 3.96 (95% CI, 1.84–7.50) cases per 1000 infants. The incidence of pertussis among infants meeting the CDC confirmed case definition was 2.47 (95% CI, .90–5.48) cases per 1000 infants, and among infants meeting the CDC probable case definition was 0.99 (95% CI, .17–3.27) cases per 1000 infants (Table 2).

**Table 2.** Incidence of Severe and Nonsevere Pertussis, Overall and by Centers for Disease Control and Prevention Diagnostic Case Criteria

Category	No. of Infants	Person-time, mo	Incidence Rate per 1000 Person-months (95% CI)	Incidence per 1000 Infants (95% CI)
All positive *Bordetella pertussis* PCR assays^a^				
All pertussis	8	6654.5	1.14 (.57–2.28)	3.96 (1.84–7.50)
Nonsevere pertussis	5	6654.5	0.75 (.31–1.81)	2.47 (.90–5.48)
Severe pertussis	3	6654.5	0.43 (.14–1.33)	1.48 (.38–4.03)
Infants meeting CDC confirmed pertussis diagnostic criteria				
All pertussis	5	6654.5	0.75 (.31–1.81)	2.47 (.90–5.48)
Nonsevere pertussis	2	6654.5	0.29 (.07–1.14)	0.99 (.17–3.27)
Severe pertussis	3	6654.5	0.43 (.14–1.33)	1.48 (.38–4.03)
Infants meeting CDC probable pertussis diagnostic criteria				
All pertussis	2	6654.5	0.29 (.07–1.14)	0.99 (.17–3.27)
Nonsevere pertussis	2	6654.5	0.29 (.07–1.14)	0.99 (.17–3.27)
Severe pertussis	0	6654.5	0.0 (NA)	0.0 (NA)

Abbreviations: CDC, Centers for Disease Control and Prevention; CI, confidence interval; NA, not applicable; PCR, polymerase chain reaction.

^a^ Any infant meeting the syndromic screening definition with a positive PCR test.

The incidence rate of pertussis according to our pertussis case definition was 1.14 (95% CI, .57–2.28) cases per 1000 person-months. The incidence rate of pertussis according to the CDC confirmed pertussis case definition was 0.75 (95% CI, .31–1.81) cases per 1000 person-months, and among infants meeting the CDC probable case definition was 0.30 (95% CI, .08–1.20) cases per 1000 person-months (Table 2).

Three cases met the severe pertussis criteria of a (modified) Preziosi score ≥7 (incidence rate of severe pertussis, 0.43 [95% CI, .14–1.33] cases per 1000 person-months), with all of these cases meeting the CDC confirmed case definition (Table 2).

**Figure 2.**
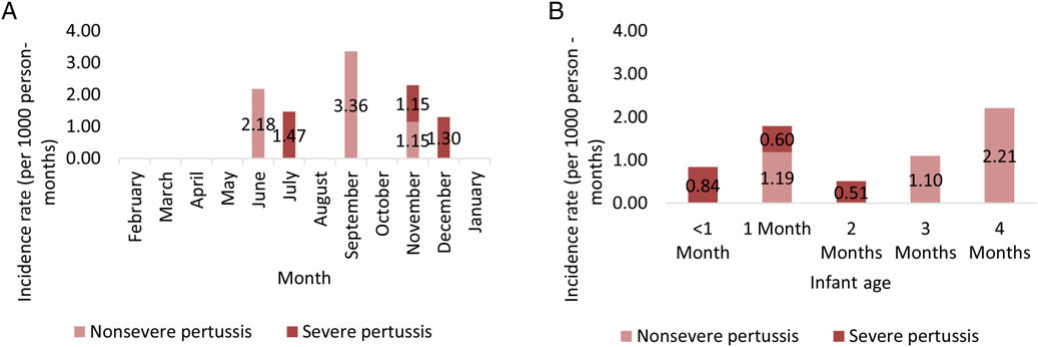
*A*, Incidence of severe and nonsevere pertussis by calendar month. *B*, Incidence of severe and nonsevere pertussis by infant age, in months. Pertussis incidence presented here includes all pertussis cases, defined as meeting the syndromic case definition plus positive polymerase chain reaction test for *Bordetella pertussis*.

Pertussis cases occurred between June and December 2015, with 1 case each in June and July, 3 cases in September, 2 cases in November, and 1 case in December. The 3 severe pertussis cases occurred in July, November, and December (Figure 2). We also evaluated pertussis cases by month of age at diagnosis, with age-specific person-time computed to estimate age-specific pertussis incidence rates.

The most common symptoms were cough (occurring in 7 of 8 cases), severe chest indrawing (6 of 8 cases), and tachypnea and coryza (4 of 8 cases for both). Additionally, 5 of the 8 cases presented with upper respiratory symptoms not otherwise specified (Supplementary Table 2). Among the 3 severe pertussis cases, there were 3 specified symptoms that were present in all cases—cough, coryza, and severe chest indrawing, whereas whoop and tachypnea were seen in 2 of the 3 severe cases. Among the 5 nonsevere cases, the most common symptoms were cough (n = 4) and severe chest indrawing (n = 3). There were no hospitalizations among the 8 pertussis cases. There was 1 death among the pertussis positive cases. This infant was diagnosed at 5 weeks of age with a nonsevere syndromic case of pertussis, and passed away the same week.

A detailed summary of demographic and case classification findings for the 8 PCR-confirmed cases is presented in Table 3. Notably, 5 pertussis cases were in male infants, and 3 in female infants, similar to the slight excess of males in the total surveillance study. The median time to diagnosis from enrollment was 6 weeks.

**Table 3.** Descriptive Summary of Demographic and Case Classification Findings for Polymerase Chain Reaction–Confirmed Pertussis Cases

ID	Sex	Age at Enrollment, wk	Age at Diagnosis, wk	Time to Diagnosis, wk	Month of Diagnosis	Modified Preziosi Scale Score	No. of Pentavalent Vaccine Doses Received	Age, wk, of Each Pentavalent Vaccine Dose Received	Case Type^a^
32311	Female	2	3	1	November	14	2	10, 15	Confirmed
30361	Male	2	6	4	July	10	2	8, 16	Confirmed
18111	Male	1	9	8	December	9	0	NA	Confirmed
42201	Male	3	13	10	September	5	1	13	Confirmed
53401	Female	2	5	3	November	6	1	13	Confirmed
50701	Male	8	18	10	June	6	0	NA	Probable
42291	Male	1	15	14	September	0	1	11	Probable
53321	Female	3	5	2	September	5	0	NA	Syndromic

Abbreviation: NA, not applicable.

^a^ “Confirmed” represents cases meeting the Centers for Disease Control and Prevention (CDC) confirmed case classification; “Probable” represents cases meeting the CDC probable case classification; “Syndromic” represents cases meeting the syndromic case definition plus polymerase chain reaction confirmation.

## DISCUSSION

Ours is one of the first prospective surveillance studies to evaluate the burden of pertussis in young infants in developing countries. We found a moderate burden of pertussis disease in our surveillance catchment area and have identified pertussis as a pathogen responsible for considerable disease among infants in Karachi. This is a first step in estimating the public health impact of pertussis in young infants in low- and lower-middle-income countries. The next logical step would be to estimate the extent to which pertussis contributes to severe disease, hospitalizations, and deaths among young infants in these low-resource settings. There are indications that at least in some other low-resource settings, such as Johannesburg, South Africa, pertussis is associated with hospitalizations of young infants (see Nunes et al in this supplement).

There are limited comparable high-quality burden data on infant pertussis from other low- and lower-middle-income countries. In a recent review of the literature, Tan et al found few published epidemiologic data from the WHO Africa, Eastern Mediterranean, Southeast Asia, and Western Pacific regions [8]. Moreover, as much of the available data are generally not collected through established surveillance systems for pertussis, incidence rates are often estimated based on retrospective studies of hospitalized infants. Nevertheless, data from high-income countries indicate that pertussis incidence is higher in disadvantaged populations. For example, Vitek et al reported that, in the 1990s, pertussis-associated mortality was at least 2.6 times higher in Hispanic infants compared with non-Hispanic infants [9]. Similarly, between 2002 and 2004, the incidence of pertussis-associated hospitalizations was 101 per 100 000 among Native American and Alaskan infants compared with 68 per 100 000 among the general US infant population [10].

There have been several attempts to estimate the global burden of pertussis. In 1999, Crowcroft et al produced a global estimate of 48.5 million cases and 295 000 deaths [11]. Later models estimated substantially lower estimates of the global burden of pertussis. For example, in 2010, Black et al estimated that 16 million cases of pertussis and 195 000 pertussis-associated deaths occurred globally in 2008 [12]. Despite these estimates, the actual reported cases have been only a fraction of estimated cases. For example, in 2014 only 139 786 cases of pertussis were reported globally [13]. There are many reasons for uncertainty in pertussis burden estimates including secular trends in surveillance intensity and approaches, evolution in diagnostic methods, changes in national vaccine schedules, vaccine products used, and cyclical trends in pertussis incidence [13–15]. However, the most significant contributor to nonrobust pertussis burden estimates is lack of data from low- and lower-middle-income countries. Studies such as ours will help fill this data gap.

Among the 8 pertussis cases in our community-based study, 3 were classified as severe based on a modified Preziosi score. Yet, none of these severe cases were hospitalized. Importantly, the original Preziosi scale was designed to identify combinations of presenting signs and symptoms that would dichotomize pertussis cases into severe and nonsevere illnesses, with the threshold defined by the median score in the study population—it was not necessarily meant to be predictive of clinical outcomes [7]. Moreover, as shown here, the age group followed in this study can present with symptoms not common among the population originally studied by Preziosi et al. Two subsequent retrospective studies were able to identify risk factors or clinical predictors of severe disease [16] (as defined by clinical outcomes) or death [17] due to pertussis, including young age (<2 months), prematurity, fever at presentation, and peak white blood cell and lymphocyte counts. However, both studies were conducted in settings (Australia and the United States) where pertussis transmission, healthcare-seeking behavior, and pediatric healthcare services may not be representative of developing countries. Prospective surveillance of hospitalized infants with suspected or confirmed pertussis in our setting would help generate evidence for a more objective pertussis severity assessment in low- and lower-middle-income countries.

The infant immunization schedule in Pakistan recommends vaccination with diphtheria, tetanus, and whole-cell pertussis (DTwP), *Haemophilus influenzae* type b, and hepatitis B at 6, 10, and 14 weeks of age [18]. Of the 3 severe pertussis cases, all were too young to be fully vaccinated. This is in line with the relative age distribution of pertussis reported from a variety of settings. This distribution of cases provides support for a maternal pertussis immunization strategy to reduce the infant pertussis burden. Moreover, our syndromic case definition, designed to be as sensitive as possible, performed as well as the standard US CDC case definition. Of the 8 PCR-confirmed pertussis cases identified to date in this surveillance cohort, 7 met the CDC pertussis case definition, including 5 that met the CDC confirmed case definition and 2 that met the CDC probable case definition.

There a few potential limitations of our study. First, the surveillance period spanned approximately 1 year, even though pertussis is known to exhibit multiyear periodicity, with cycles occurring every 2–4 years [19]. Hence, our findings should be interpreted as a snapshot of pertussis epidemiology in an urban, South Asian population. Moreover, our study was conducted in a setting with low whole-cell pertussis vaccine (DTwP) coverage. The estimated 3-dose DTwP coverage at our study sites is 40%–50%, as measured by the demographic and surveillance system at these sites. This could ostensibly limit the generalizability of our findings to populations with suboptimal infant DTwP coverage. However, such populations form a substantial portion of the birth cohorts in low-income settings. Moreover, given that there is evidence that national immunization programs tend to overestimate vaccine coverage, our coverage estimates are likely to be closer to the “real” coverage. Hence, our findings are likely to be generalizable to large parts of developing country populations. Additionally, many surveillance studies have limitations due to healthcare-seeking behavior in the community under surveillance. However, given our intensive surveillance, it is unlikely that healthcare-seeking behavior had an impact on estimates of pertussis incidence in our study.

In conclusion, while the current study established that pertussis is a cause of disease in early infancy in a low-income South Asian setting, there is a need to better characterize the burden of pertussis in hospitalized cases. Moreover, given that pertussis often has multiyear cycles, the next steps could include continuing surveillance with an emphasis on identifying severe disease in hospitalized infants.

## Supplementary Data

Supplementary materials are available at http://cid.oxfordjournals.org. Consisting of data provided by the author to benefit the reader, the posted materials are not copyedited and are the sole responsibility of the author, so questions or comments should be addressed to the author.

Supplementary Data
