# Supplementary material for: Epidemiology of Pertussis Among Young Pakistani Infants: A Community-Based Prospective Surveillance Study
Source: Clin Infect Dis. 2016 Nov 2;63(Suppl 4):S148–53. doi: 10.1093/cid/ciw561 (PMC5106628; doi:10.1093/cid/ciw561)
Supplement: Supplementary Data [file supp_63_suppl-4_S148__index.html]

Supplementary Data 

# Epidemiology of Pertussis Among Young Pakistani Infants: A Community-Based Prospective Surveillance Study

## Supplementary Data

Supplementary Data

- Supplementary Data - Docx file
