# Supplementary material for: Epidemiology of Pertussis Among Young Pakistani Infants: A Community-Based Prospective Surveillance Study
Source: Clin Infect Dis. 2016 Nov 2;63(Suppl 4):S148–53. doi: 10.1093/cid/ciw561 (PMC5106628; doi:10.1093/cid/ciw561)
Supplement: Supplementary Data [file supp_ciw561_ciw561supp.docx]

**Supplemental Material**

| **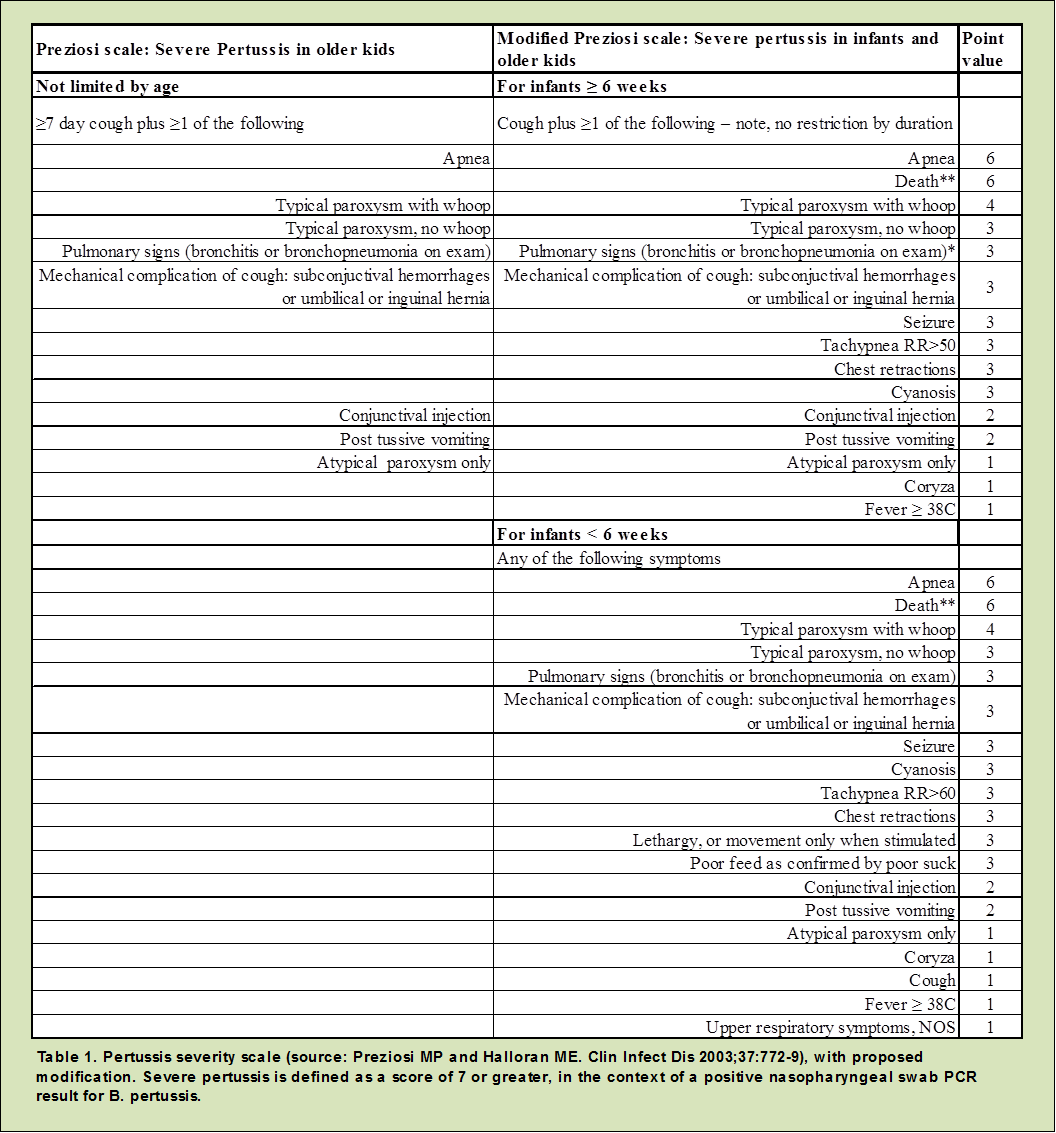**  **Figure S1. Pertussis severity scale (source: Preziosi MP and Halloran ME. Clin Infect Dis 2003;37:772-9), with proposed modification. Severe pertussis is defined as a score of 7 or greater in the context of a positive nasopharyngeal swab PCR results for *B. pertussis.*** |
| --- |

| **Table S1.** Baseline characteristics of mothers | |
| --- | --- |
| **Maternal Demographic Information** |  |
| Median age in years (IQR)* (N = 197) | 22 (26 – 30) |
| Median gestational age in months (IQR)* (N = 197) | 34 (32 – 37) |
| Enrolled at time of birth (N (%))* | 65 (29.4%) |
| *Highest level of maternal education (N = 2021)* | **N (%)** |
| 0 years | 1,078 (54.0) |
| 1 to 5 years | 414 (20.7) |
| 6 to 9 years | 222 (11.2) |
| 10 years or more | 283 (14.2) |
| Missing | 24 |
| *Highest level of paternal education (N = 2021)* |  |
| 0 years | 939 (47.0) |
| 1 to 5 years | 377 (18.9) |
| 6 to 9 years | 279 (14.0) |
| 10 years or more | 402 (20.1) |
| Missing | 24 |
| *Vaccines During Pregnancy*(N = 197)* |  |
| Ever received TT vaccination? (N (%)) | 113 (57.4) |
| Median number of doses of tetanus toxoid (IQR) | 2 (1 – 2) |
| * Specific to mothers enrolled in the closed cohort (N = 221) only. | |

| **Table S2.** Descriptive Summary of Symptoms for Eight Infants Meeting the Syndromic Case Definition with PCR Confirmation. | | | | | | | | | |
| --- | --- | --- | --- | --- | --- | --- | --- | --- | --- |
| **Subject identification number:** | **32311** | **30361** | **18111** | **42201** | **53401** | **50701** | **42291** | **53321** |  |
| Case classification* | Conf. | Conf. | Conf. | Conf. | Conf. | Prob. | Prob. | Synd. |  |
| Age (weeks) at diagnosis | 3 | 6 | 9 | 13 | 5 | 18 | 15 | 5 |  |
| *Modified Preziosi Score* | 14 | 10 | 9 | 5 | 6 | 6 | 0 | 4 | **Totals** |
| Cough/paroxysms | Yes | Yes | Yes | Yes | Yes | Yes | Yes | No | 87.5% (7/8) |
| Cough at least 2 weeks | Yes | Yes | Yes | Yes | Yes | No | No | No | 62.5% (5/8) |
| Coryza | Yes | Yes | Yes | No | Yes | No | No | Yes | 50.0% (4/8) |
| Whoop | Yes | Yes | No | No | No | No | No | No | 25.0% (2/8) |
| Apnea | No | No | No | No | No | No | No | No | 0.0% (0/8) |
| Post-tussive emesis | No | No | Yes | Yes | No | No | No | No | 25.0% (2/8) |
| Cyanosis | No | No | No | No | No | No | No | No | 0.0% (0/8) |
| Seizure | No | No | No | No | No | No | No | No | 0.0% (0/8) |
| Tachypnea | No | Yes | Yes | Yes | No | Yes | No | No | 50.0% (4/8) |
| Severe chest indrawing | Yes | Yes | Yes | No | Yes | Yes | No | Yes | 75.0% (6/8) |
| Movement only when stimulated | No | No | No | No | No | No | No | No | 0.0% (0/8) |
| Poor feeding confirmed by poor suck | Yes | No | No | No | No | No | No | No | 0.0% (0/8) |
| Close exposure to a family members with prolonged afebrile cough illness | No | No | No | No | Yes | No | No | No | 12.5% (1/8) |
| Fever | No | No | No | No | No | No | No | No | 0.0% (0/8) |
| Wheeze | No | Yes | No | No | No | Yes | No | No | 25.0% (2/8) |
| Mechanical complications of cough | No | No | No | No | No | No | No | No | 0.0% (0/8) |
| Conjunctivial injection | No | No | No | No | No | No | No | No | 0.0% (0/8) |
| Atypical paroxysms only | Yes | No | No | No | Yes | No | No | No | 25.0% (2/8) |
| Upper respiratory symptoms NOS | Yes | Yes | Yes | Yes | No | No | Yes | No | 62.5% (5/8) |
| Other | No | No | No | No | No | No | No | Yes | 12.5% (1/8) |
| * Conf. represents cases meeting the CDC confirmed case classification; Prob. represents cases meeting the CDC probably case classification; Synd. represents cases meeting the syndromic case definition plus PCR confirmation. | | | | | | | | | |

**Outcome definitions.**

1. Infant meets the syndromic definition and has a positive PCR test for *B. pertussis*. (PrePY definition)
2. Infant meets the US Centers for Disease Control and Prevention case definition of
   1. Probable case
      1. Acute cough illness of any duration, with at least one of the following symptoms:
         1. Paroxysms of coughing
         2. Inspiratory whoop
         3. Posttussive vomiting
         4. Apnea (with or without cyanosis)
      2. Meets one of the following criteria
         1. PCR positive test for pertussis
         2. Contact with a laboratory-confirmed case of pertussis
   2. Confirmed case
      1. Meets the clinical case definition of cough illness of 2 or more weeks with at least one of the following symptoms, accompanied by positive PCR test for pertussis or contact with a laboratory-confirmed case of pertussis:
         1. Paroxysms of coughing
         2. Inspiratory whoop
         3. Posttussive vomiting
         4. Apnea (with or without cyanosis)
      2. Acute cough of any duration with isolation of *B. pertussis* from a clinical specimen
